# *Deinococcus radiodurans* Toxin–Antitoxin MazEF-dr Mediates Cell Death in Response to DNA Damage Stress

**DOI:** 10.3389/fmicb.2017.01427

**Published:** 2017-07-26

**Authors:** Tao Li, Yulan Weng, Xiaoqiong Ma, Bing Tian, Shang Dai, Ye Jin, Mengjia Liu, Jiulong Li, Jiangliu Yu, Yuejin Hua

**Affiliations:** ^1^Key Laboratory of Nuclear Agricultural Science of Ministry of Agriculture and Zhejiang Province, Institute of Nuclear-Agricultural Sciences, Zhejiang University Hangzhou, China; ^2^Central Laboratory, The First Affiliated Hospital of Zhejiang Chinese Medical University Hangzhou, China

**Keywords:** toxin–antitoxin system, MazEF, *Deinococcus radiodurans*, cell death, DNA damage stress

## Abstract

Here we identified a functional MazEF-dr system in the exceptionally stress-resistant bacterium *D. radiodurans*. We showed that overexpression of the toxin MazF-dr inhibited the growth of *Escherichia coli*. The toxic effect of MazF-dr was due to its sequence-specific endoribonuclease activity on RNAs containing a consensus 5′ACA3′, and it could be neutralized by MazE-dr. The MazF-dr showed a special cleavage preference for the nucleotide present before the ACA sequence with the order by U>A>G>C. MazEF-dr mediated the death of *D. radiodurans* cells under sub-lethal dose of stresses. The characteristics of programmed cell death (PCD) including membrane blebbing, loss of membrane integrity and cytoplasm condensation occurred in a fraction of the wild-type population at sub-lethal concentration of the DNA damaging agent mitomycin C (MMC); however, a MazEF-dr mutation relieved the cell death, suggesting that MazEF-dr mediated cell death through its endoribonuclease activity in response to DNA damage stress. The MazEF-dr-mediated cell death of a fraction of the population might serve as a survival strategy for the remaining population of *D. radiodurans* under DNA damage stress.

## Introduction

Toxin–antitoxin (TA) systems, also referred to as addiction or suicide modules, are distributed widely in free-living organisms including bacteria and archaea (Pandey and Gerdes, 2005; Makarova et al., 2009). TA systems have attracted increasing attention in recent years because of the effects they exhibit during bacterial responses to environmental stresses and infection. Typical TA systems have two components that are co-transcribed from a bi-cistronic operon, in which the upstream gene encodes a labile antitoxin and the downstream gene encodes a stable toxin. TA systems are currently classified into at least three types: types I and III antitoxins are RNAs that either inhibit the expression or activity of the toxin, while the most widespread type II TA system contains a protein antitoxin. In type II TA systems, the antitoxin neutralizes the activity of the toxin via a physical interaction (Kamada et al., 2003). Once the concentration of the antitoxin decreases, the free toxin will act on its targets (RNAs or proteins), which results in bactericidal or bacteriostatic effects. In addition, most of the reported type II TA systems share a negative auto-regulation mechanism (Marianovsky et al., 2001). Type II TA systems discovered originally in plasmids were low-copied, which are responsible for a process called post-segregational killing (Ogura and Hiraga, 1983; Gerdes et al., 1986). In contrast to plasmidic TA systems, chromosome-bearing TA systems are more complicated due to their multi-copy nature.

The MazEF system, a chromosome-bearing type II TA system, was first identified in *Escherichia coli* (Aizenman et al., 1996). The toxin MazF is also known as mRNA interferase which usually cleaves mRNAs at specific sites (Nariya and Inouye, 2008). Recently, 16S rRNA, 23S rRNA and tRNAs were identified as the substrates of MazF in *E. coli* and *Mycobacterium tuberculosis* (Vesper et al., 2011; Schifano et al., 2013, 2014). MazF was involved in biofilm formation of *E. coli* (Kolodkin-Gal et al., 2009; Han et al., 2010; Tripathi et al., 2014). Aizenman et al. (1996), Hazan et al. (2004), Engelberg-Kulka et al. (2006) reported the MazF-centric programmed cell death (PCD) system in *E. coli* could be activated by various stress conditions, including amino acid starvation, antibiotics, oxidative stress and high temperature. The PCD process has also been observed in *Myxococcus xanthus*, in which MazF-mediated cell death is required for the development of multicellular fruiting body (Nariya and Inouye, 2008). The exact physiological functions and mechanisms of the MazEF system remain unclear.

*Deinococcus radiodurans* belongs to the phylum Deinococcus–Thermus extremophiles. *D. radiodurans* is an ideal organism for studying bacterial mechanisms in response to environmental stresses because of its exceptional tolerance against DNA damaging agents including ionizing radiation, ultraviolet radiation, oxidative stress and mitomycin C (MMC). The viable fractions of *D. radiodurans* in response to stresses exhibited a characteristic sigmoid dose-response curve with a “shoulder” representing resistance at low doses and specially a “steep slope” representing substantial decrease of viability at sub-lethal and higher doses. The sigmoid dose-response curve distinguishes the extremophiles from damage-sensitive bacteria such as *E. coli* with the exponential dose-response curve (Slade and Radman, 2011). Several factors and mechanisms have been suggested for the *D. radiodurans* survival under stresses (Zahradka et al., 2006; Slade and Radman, 2011; Pavlopoulou et al., 2016): First, strong cell defense systems, including catalase and Mn^2+^-dependent antioxidant enzymes (superoxide dismutase and low-molecular-weight Mn^2+^-metabolite complexes), protect essential proteins such as DNA repair enzymes from oxidative damage. Second, efficient DNA repair mechanisms are required for the recovery of a fully functional genome, for instance, RecA and PPrI (IrrE) are critical repair proteins in homologous recombination pathway of *D. radiodurans*. Moreover, a combined mechanism including efficient DNA, protein, membrane lipid protection and repair was proposed to be required for the organism’s response to extreme radiation (Pavlopoulou et al., 2016). However, the detailed survival mechanism from the view of a population of *D. radiodurans* in response to stresses is far from being understood.

Here the *dr0416-dr0417* locus of *D. radiodurans* was identified as encoding a functional MazEF system. We investigated the biochemical activities and properties of the MazEF-dr system to determine the functions of TA system. The preferential RNA sequence recognized by MazEF-dr and the roles of MazEF-dr in response to DNA damaging agents were also investigated to elucidate the TA mechanism.

## Results

### Identification of Chromosomal *mazEF* Modules in *D. radiodurans*

The genome of *D. radiodurans* R1 was screened for genes encoding MazEF homologs by the BlastP program^1^ using established *E. coli* MazEF (EcMazEF) as query sequences. Two pairs of putative MazEF-encoding genes were found in the *D. radiodurans* genome: *dr0416-dr0417* and *dr0661-dr0662*, which are consistent with the predicted sequences in the Toxin-Antitoxin Database (TADB) (Shao et al., 2011). DR0417 and DR0662 share 46 and 29% identities, respectively, to *E. coli* MazF, indicating that *dr0417* and *dr0662* might encode the toxin proteins. The *dr0416* and *dr0661*, which are upstream of *dr0417* and *dr0662*, respectively, might encode the antitoxin. DR0417 contains several conserved amino acid residues, such as Gly22, Arg29, Pro30, and Pro59 (Supplementary Figure S1), which are required for substrate binding and toxin activity (Simanshu et al., 2013), while DR0662 showed less conservation in the substrate binding sites, e.g., Ala21 and Asp59 (Supplementary Figure S1).

**FIGURE 1:**
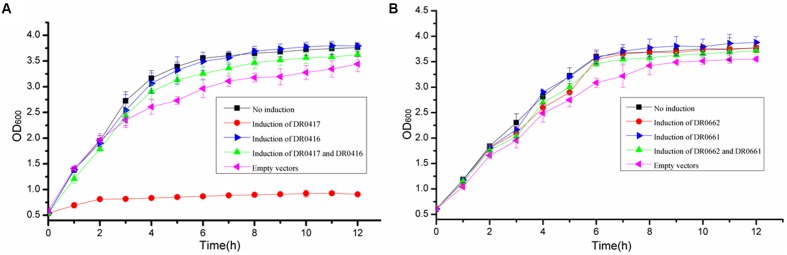
Effects of over-expression of *dr0416-dr0417* or *dr0661-dr0662* on the growth of *Escherichia coli*. **(A)** Growth curve of *E. coli* BW25113Δ6 cells harboring *dr0416*-pET28a and/or *dr0417*-pBAD33 after induction by IPTG and arabinose, respectively. The cells harboring both *dr0416*-pET28a and *dr0417*-pBAD33 in the absence of induction (black line, No induction), *dr0417*-pBAD33 in the presence of arabinose induction (red line), *dr0416*-pET28a in the presence of IPTG induction (blue line), or both the *dr0416*-pET28a and *dr0417*-pBAD33 in the presence of IPTG and arabinose induction (green line) are shown. The cells harboring only empty plasmids were used as the control (pink line, Empty vectors). **(B)** Growth curve of *E. coli* BW25113Δ6 cells harboring *dr0661*-pET28a and/or *dr0662*-pBAD33 induced by IPTG and arabinose, respectively. The cells harboring both the *dr0661*-pET28a and *dr0662*-pBAD33 in the absence of induction (black line, No induction), *dr0662*-pBAD33 in the presence of arabinose induction (red line), *dr0661*-pET28a in the presence of IPTG induction (blue line), or both the *dr0661*-pET28a and *dr0662*-pBAD33 in the presence of IPTG and arabinose induction (green line) are shown. The cells harboring only empty plasmids were used as the control (pink line, Empty vectors). The OD_600_ was monitored.

To determine whether two pairs of MazEF homologs are functional TA systems, each MazEF homology module was overexpressed in *E. coli*, and the inhibition of cell growth was monitored. Western blot assays with anti-DR0417 and anti-DR0662 antibodies confirmed that the proteins were expressed successfully in *E. coli* (Supplementary Figure S2). *E. coli* with overexpression of DR0417 exhibited substantial cell growth defect compared with the control cells containing only empty vectors (**Figure 1A**). In contrast, cells with co-overexpression of DR0416 and DR0417 or those with over-expression of DR0416 alone showed similar growth profiles to that of the control, indicating that the DR0417-induced growth defect could be neutralized by DR0416 (**Figure 1A**). However, over-expression of DR0662 and/or DR0061 in *E. coli* did not result in obvious changes of cell growth (**Figure 1B**). These results indicated that the *dr0416-dr0417* locus may function as MazEF-type TA system and we therefore refer to them as MazEF-dr for MazEF in *D. radiodurans*.

### Biochemical Characterization of MazEF-dr

**FIGURE 2:**
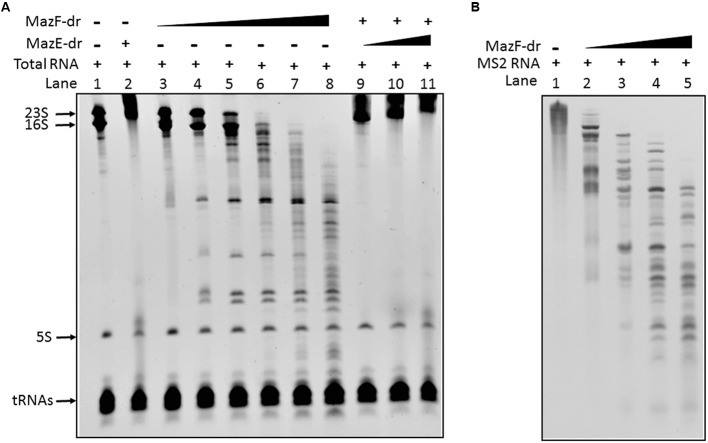
*In vitro* endoribonuclease activity of MazF-dr is neutralized by MazE-dr. **(A)** 2 μg of total RNA from *D. radiodurans* was used in each reaction. Lane 1, the control containing RNA only. Lane 2, RNA products following incubation with MazE-dr (100 nM). Lanes 3–8, RNA products following incubation with MazF-dr at increasing concentrations ranging from 1 to 100 nM. Lanes 9–11, RNA products from a reaction with 50 nM MazF-dr that was pre-incubated with MazE-dr (50–300 nM). **(B)** 0.1 μg of MS2 RNA was incubated with MazF-dr at increasing concentrations ranging from 0 to 0.3 μM for 15 min (Lanes 1–5). The RNA products were separated using a 4.5% Urea-PAGE gel and stained by EtBr.

To evaluate the biochemical properties of MazEF-dr, the *in vitro* activity of MazF-dr was assessed. Total cellular RNAs were extracted from *D. radiodurans* cells. Following incubation with purified MazF-dr, a gradual accumulation of cleavage products appeared, and they increased with increasing protein concentration (**Figure 2A**). Especially 16S rRNA and 23S rRNA of *D. radiodurans* were cleaved completely into smaller fragments by MazF-dr at high concentration (100 nM, lane 8 in **Figure 2A**), while few cleavage products of 5S rRNA and tRNAs were detected. When an exogenous RNA (MS2 RNA, RefSeq accession no. NC_001417) was used as a substrate of MazF-dr, a specific cleavage profile was also observed (**Figure 2B**). Thus, MazF-dr seems to be an endoribonuclease that cleaves RNAs. However, pre-incubation of MazF-dr with MazE-dr completely inhibited the RNase activity of MazF-dr (lanes 9–11 in **Figure 2A**), indicating that the activity of MazF-dr could be neutralized by MazE-dr. These results suggest that MazF-dr acts as the toxin and MazE-dr is the antitoxin that might abrogate the toxic effect of MazF-dr.

### Conserved Cleavage Site of RNA by MazF-dr

**FIGURE 3:**
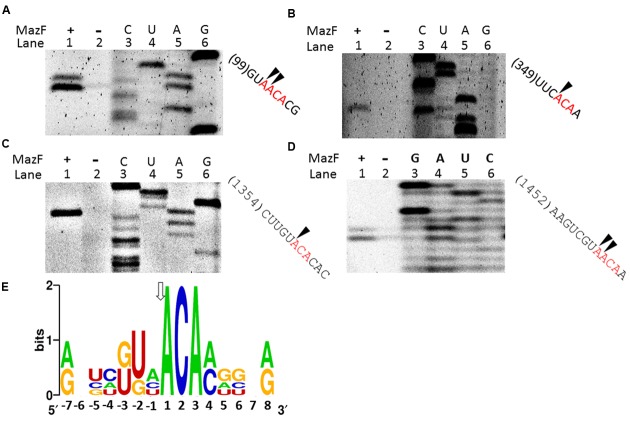
Conserved cleavage sites of MazF-dr in RNAs. **(A–D)** Primer extension experiments using 5′-FAM labeled primers following the cleavage of synthetic 16S rRNA by MazF-dr. Lane 1, 16S rRNA incubated with MazF-dr; lane 2, a control reaction in the absence of MazF-dr; lanes 3–6, sequencing ladder. Sites cleaved by MazF-dr were determined, and indicated by arrows on the corresponding RNA sequences listed on the right. The same oligonucleotide was used for both the primer extension and sequencing reactions. All data are representative of three independent experiments. **(E)** Sequence logo plot of a multiple sequence alignment of RNA sequences flanking the indicated cleavage sites in 16S RNA by MazF-dr. The height of a nucleotide corresponds to its conservation in the multiple-sequence alignment. Numbering reflects the nucleotide position relative to the major cleavage site, as indicated by an arrow.

A primer extension assay was used to analyze the RNA cleavage site of MazF-dr. 16S rRNA was used as the substrate to determine the cleavage site of MazF-dr. As shown in **Figures 3A–D**, two cleavage patterns were identified, both of which consisted of an ACA consensus sequence (**Figure 3E**). In the first pattern (**Figures 3A,D**), cleavage occurred at the 5′ end of an adjacent A in the tetrad sequence (^↓^A^↓^ACA). For the other cleavage pattern (**Figures 3B,C**), cleavage occurred at the 5′ end of the A (^↓^ACA). Notably, the cleavage activity of MazF-dr at the A^↓^ACA site was higher than that at the ^↓^AACA site, as determined by the product intensity, suggesting that the ^↓^ACA is the major cleavage site.

**FIGURE 4:**
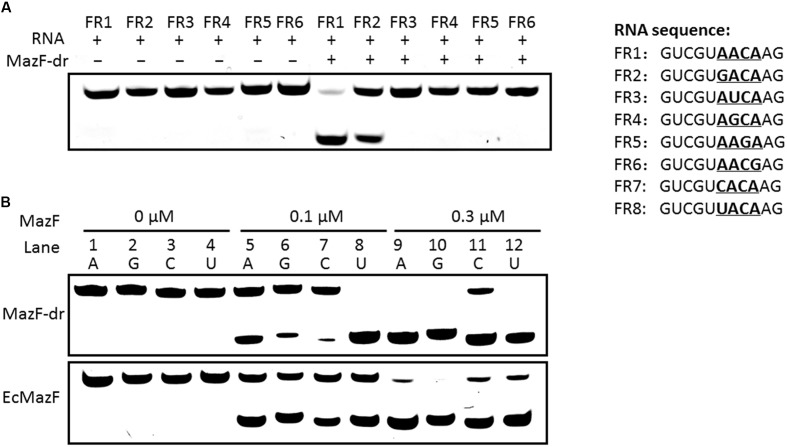
Cleavage activity of MazF-dr on synthesized RNA derivatives. **(A)** MazF-dr cannot cleave RNAs lacking the ACA consensus sequence. Synthesized RNA derivatives containing the ACA sequence (FR1 and FR2) or not (FR3–6) were used as the substrates of MazF-dr. Each RNA oligo labeled with 5′ FAM was incubated with or without MazF-dr for 15 min. **(B)** Comparison of the cleavage activities of MazF-dr and EcMazF using different substrates in which the nucleotide before the ACA sequence was substituted with A (FR1, lanes 1, 5, and 9), G (FR2, lanes 2, 6, and 10), C (FR7, lanes 3, 7, and 11) or U (FR8, lanes 4, 8, and 12). MazF-dr had the highest cleavage activity on the substitution of U in comparison with the A, G, or C substitutions, whereas EcMazF did not demonstrated obvious preference on the nucleotide substitution. The oligonucleotides listed in the right panel were incubated with MazF-dr at the increasing concentrations ranging from 0 to 0.3 μM. The fluorescently labeled RNA products were separated by 20% urea-PAGE. All data are the representative of three independent experiments.

To further validate the consensus cleavage sequence and the effects of the 5′ terminal nucleotide adjacent to the ACA sequence of substrates on the activity of MazF-dr, we synthesized a series of 5′ fluorescein amidite (FAM)-labeled RNA derivatives (Supplementary Table S2) and measured the cleavage activity of MazF-dr on these RNAs. As shown in **Figure 4A**, MazF no longer cleaved the RNAs that lacked the ACA consensus sequence. These results indicated that MazF-dr specifically cleaved RNAs containing a consensus 5′ACA3′ sequence in a ribosome-independent fashion. In addition, substitutions of the nucleotide before the ACA sequence with A, G, U, or C did not prevent cleavage function of MazF-dr, but it affected the cleavage activity of MazF-dr (**Figure 4B**). MazF-dr had the highest cleavage activity on the U-substituted sequence, showing a decreasing cleavage activity in the order of U>A>G>C, suggesting that the surrounding nucleotide composition of the substrates has the impact on the activity of the toxin. Unlike *D. radiodurans* MazF, EcMazF did not have a cleavage preference for the nucleotide before the ACA consensus sequence (**Figure 4B**).

### MazEF-Mediated Cell Death of *D. radiodurans* under DNA Damage Stresses

**FIGURE 5:**
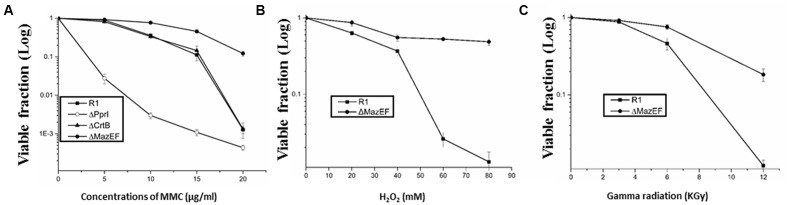
Effects of DNA damage stress on cell viability. Cell viability of the *D. radiodurans* wild-type (-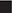-) and ΔMazEF-dr (-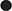-) strains following exposure to different doses of MMC **(A)**, H_2_O_2_ **(B)**, and γ-radiation **(C)**. Mutants of a DNA repair related gene (*pprI*) and an antioxidant related gene (*crtB*) were used in **(A)** for comparison purpose. Cell viability was determined by comparing the number of colonies of treated cells with that of untreated cells incubated on plates at 30°C overnight. Values represent the mean ± standard deviation of three independent experiments.

We evaluated the roles of the *mazEF-dr* module in *D. radiodurans* in response to extreme stresses. A double gene knockout mutant of *mazEF-dr*, designated ΔMazEF-dr, was constructed (Supplementary Figure S3). The growth curve of the ΔMazEF-dr did not differ significantly from that of the wild-type strain under stress-free condition (data not shown), indicating that MazEF-dr is not essential for normal bacterial growth. However, the viability of ΔMazEF-dr differed greatly from that of the wild type under different doses of DNA damage stresses, including MMC, H_2_O_2_, and γ-radiation (**Figures 5A–C**). Under low doses of DNA damaging agents (<5 μg/ml MMC, <40 mM H_2_O_2_, <6 kGy γ-ray), the dose-response curve of the wild type and ΔMazEF-dr showed a similar “shoulder,” representing little decrease in the viability; however, a “steep slope” representing a substantial decrease of the viable fraction of the wild-type strain was observed at higher doses of the DNA damage agents (10–15 μg/mL MMC, 40–80 mM H_2_O_2_, 6 kGy γ-ray), compared with ΔMazEF-dr cells (**Figures 5A–C**). Mutants of a DNA repair related gene (*pprI*) and an antioxidant related gene (*crtB*), which was involved in the carotenoid biosynthesis, were used for the comparison purpose (**Figure 5A**). The *pprI* mutant was more sensitive to MMC stress than those of the wild type and ΔMazEF-dr, while the *crtB* mutant was as resistant as the wild type.

**FIGURE 6:**
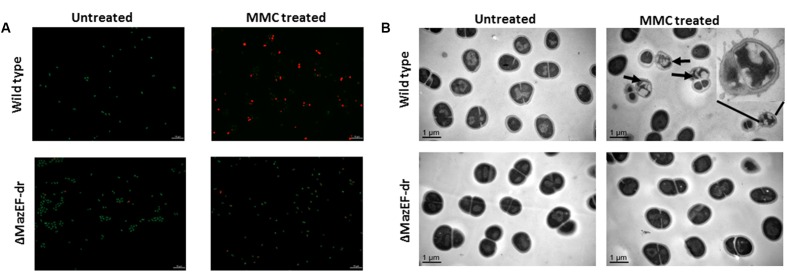
Cell death mediated by MazEF-dr. **(A)** Confocal microscopy of *D. radiodurans* wild-type and Δ*mazEF-dr* cells treated with or without a sub-lethal MMC concentration (15 μg/ml for 40 min). Cell viability was detected by the Live/Dead Kit containing PI and SYTO 9 (Invitrogen). Living cells were stained by SYTO 9 (green) and death cells were stained by PI (red). **(B)** TEM images of *D. radiodurans* wild-type and Δ*mazEF-dr* cells treated with or without a sub-lethal MMC concentrations (15 μg/ml for 40 min). Arrows indicate the cell death characteristics of partial members of the wild-type cells including loss of membrane integrity, membrane blebbing and cytoplasm condensation. The inset diagram in **(B)** shows an amplified dead cell. Scale bars correspond to 1 μm. All data are the representative of three independent experiments.

The cell viability data are consistent with the cell phenotypes obtained by a confocal microscopy assessment using the Live/Dead Kit (**Figure 6A**). The living cells were stained by SYTO 9 (green) and the dead cells were stained by propidium iodide (PI) (red). Treatment with a sub-lethal dose of MMC (15 μg/ml) led to the death of a substantial number of the wild-type cells, while most of the mutant cells remained alive after the treatment. A transmission electron microscopy (TEM) analysis demonstrated that morphological changes occurred in 24% (calculated from three independent views) of the wild-type cells, compared with the mutant cells under sub-lethal dose of the MMC stress (**Figure 6B**), including the loss of membrane integrity, membrane blebbing and cytoplasm condensation, which are the characteristics of PCD (Dewachter et al., 2015). The mutation of *mazEF-dr* relieved these cell death phenotypes (**Figures 6A,B**), suggesting that the MazEF-dr mediated cell death of a fraction of the population in response to sub-lethal dose of DNA damage stress.

## Discussion

Here we identified and characterized the MazEF system in the extremophile *D. radiodurans*. Several lines of evidence demonstrated that *dr0417* encodes the toxin MazF-dr and the upstream gene *dr0416* encodes the antitoxin MazE-dr, which abolished the toxic effect of MazF-dr. First, DR0417 contains several conserved residues, which are required for the substrate binding and toxin activity (Simanshu et al., 2013). Second, overexpression of DR0417 led to a substantial growth inhibition of *E. coli* cells, and its toxic effect was relieved by overexpression of DR0416. Third, *dr0417* was confirmed to encode an endoribonuclease that cleaved RNA at specific sites, which can be neutralized by the product of *dr0416*. Furthermore, the *dr0416-dr0417* genes are co-transcribed (Supplementary Figure S4). DR0416 can bind to the promoter of the *dr0416-dr0417* operon in the presence or absence of DR0417 (Supplementary Figure S5), which is consistent with the *E. coli* MazE (Marianovsky et al., 2001).

MazF toxins often recognize a sequence containing three, five, or seven bases as the conserved recognition sites. The *E. coli* MazF specifically cleaves RNA at the 5′A^↓^CA3′ triplet sequence (where the arrow indicates the cleavage site) (Zhang et al., 2003), and MazF-mt3 and MazF-mt7 from *M*. *tuberculosis* cleave RNAs at 5′UU^↓^CCU3′ or 5′CU^↓^CCU3′, and 5′U^↓^CGCU3′, respectively (Zhu et al., 2008), while MazF from *Haloquadra walsbyi* selectively recognizes the seven-base sequence 5′UU^↓^ACUCA3′ (Yamaguchi et al., 2012). The conserved recognition site of MazF-dr is 5′ACA3′, consistent with that of *E. coli*. The ACA sequences are present in more than 97% of the mRNAs and all of the rRNAs in *D. radiodurans*, indicating that the possible targets of MazF-dr are distributed widely. Recently, Schifano et al. (2016) found that two tRNAs were the principal targets of MazF-mt9 from *M. tuberculosis* (Schifano et al., 2016). In our present study, both 23S rRNA and 16S rRNA were cleaved by MazF-dr *in vitro*, and likely inhibited the assembly of ribosomes thereby negatively impacting the translation. However, tRNAs were not affected, probably because their secondary structure abrogated the recognition and cleavage by MazF-dr.

Substitution of the 5′ terminal nucleotide before the ACA sequence with A, G, U, or C did not prevent the cleavage function of MazF-dr, but it did affect the cleavage activity of MazF-dr, which showed the highest cleavage activity on the RNA with the U substitution. In contrast, the *E. coli* MazF cleaved RNAs without an obvious preference for the nucleotide preceding the consensus ACA sequence. These results indicate that MazF-dr has a special substrate-preference that depends on the surrounding nucleotide composition of the substrates. The roles of the substrate-preference of MazF played in response to DNA damage stress are still unclear.

*mazEF* modules are involved in stress-response of some pathogens, but the mechanism by which bacteria deal with stresses is still unclear (Amitai et al., 2004; Hazan et al., 2004; Tripathi et al., 2014; Ramisetty et al., 2016). The viability curve of the wild-type *D*. *radiodurans* under MMC, H_2_O_2_, and γ-ray stresses showed a “shoulder” which indicates a resistance at low doses and specially a substantial decrease (a “steep slope”) above sub-lethal doses, which are characteristics of the sigmoid dose-response curve of extremophiles. The sigmoid dose-response curve and distinguishes the extremophiles from damage-sensitive bacteria with the exponential dose-response curve (Slade and Radman, 2011). The question was raised that whether there are internal contributors to cause mortality of *D. radiodurans* apart from the direct lethal effects of damage-causing agents on cellular components. Herein, the characteristics of cell death including membrane blebbing, loss of membrane integrity and cytoplasm condensation were observed in a fraction of the wild-type population at sub-lethal concentration of MMC treatment, thereby pointing to the physiological changes that are markers of PCD (Dewachter et al., 2015). In contrast to the wild-type cells, the cell death phenotypes were not present in the *mazEF-dr* mutant. The *mazEF-dr* mutant showed an identical cell configuration to the untreated wild type (**Figure 6**), suggesting that MazEF-dr mediates the suicide of a subpopulation and the survival of the remaining population under the stress. MazEF-dr-mediated cell death of a fraction of the population provides an explanation for the canonical sigmoid dose-response curve. The expression levels of DNA repair-related genes, including *recA* and *ddrO*, did not changed significantly under the sublethal dose of MMC (Supplementary Figure S6). We did not rule out the possibility of interactions of MazEF with other cell response systems or genes. Under extreme stressful condition of MMC (20 μg/mL), the viability of ΔMazEF-dr decreased. It is still unclear whether the mutant of *mazEF-dr* died because of the induction of some other cell death pathways or through the direct killing effect of DNA damage agents on certain essential cell components under the extreme MMC stress condition.

The MazEF system was previously reported to be activated and involved in the cell death of *E. coli* strain MC4100 under stressful conditions, such as nutrient starvation and DNA damage (Sat et al., 2001; Sat et al., 2003; Hazan et al., 2004). *D. radiodurans* is unable to use ammonia as a nitrogen source, and instead, amino acids have been shown to serve as a nitrogen source and they are also a preferred primary carbon energy source (Venkateswaran et al., 2000). *D. radiodurans* limits its biosynthetic demands under stress and imports nutrients including amino acids derived from the surrounding cultures (Slade and Radman, 2011). In the present study, the MazEF-dr-mediated death of a fraction of cells with the increasing degree of stress might be a form of “nutritional altruism” to save nutrients from the media or provide nutrients released from the dead cells to enable the survival of the remaining population. The cell suicide behavior might favor the bacterial population; however, the detailed mechanism, including whether MazF-dr kills cells randomly or induces the cell death of seriously damaged cells, is still unknown. The surviving subpopulation might become the “seed” for the renewed population when the stressful conditions are lessened. A linear pentapeptide called EDF was identified as a quorum-sensing factor that is required for the activation of the MazF-mediated cell death in *E. coli* (Kolodkin-Gal et al., 2007). We did not detect the EDF-like signal molecule in *D. radiodurans*, but we identified *N*-acyl-L-homoserine lactones (AHLs) as the quorum-sensing signals employed by *D. radiodurans*, which are induced upon cell exposure to high levels of oxidative stress (Lin et al., 2016). Generally, MazEF systems were activated when MazE was degraded by proteases such as Lon and Clp. *D. radiodurans* encodes an unusually high number of putative proteases (Makarova et al., 2001), and several of these enzymes are induced after the exposure to γ-irradiation (Tanaka et al., 2004). The activation mechanism of MazEF-dr and its possible interaction with other cellular pathways in *D. radiodurans* requires further investigations.

In conclusion, the MazEF-dr system is employed by the stress-resistant model organism *D. radiodurans* under DNA damage stress. Further studies on the induction and detailed mechanisms of the MazEF-mediated cell death pathway are required. Our findings not only provide new insights into the properties and roles of the MazEF system that is involved in the dose-response of this extremophile under DNA damage stress, they may also broaden our understanding of the importance of MazEF in modulating the behavior of a bacterial population.

## Materials and Methods

### Strains, Plasmids and Bacterial Growth Conditions

The bacterial strains and plasmids used in this study are listed in Supplementary Table S1. The *E. coli* strains DH5α and BL21 (λDE3) pLysS were used for cloning and protein expression studies, respectively. *E. coli* BW25113Δ6 (kindly provided by Prof. Nancy A. Woychik) was used for cell growth inhibition experiments. *E. coli* strains were cultured in Luria-Bertani (LB) broth or LB agar at 37°C unless otherwise noted. The media were supplemented with chloramphenicol (Cm; 15 μg/ml) or kanamycin (Kan; 40 μg/ml) if necessary. *D. radiodurans* and its derivatives were cultured in TGY (0.5% Bacto tryptone, 0.1% glucose and 0.3 Bacto yeast extract) broth or TGY agar at 30°C. Streptomycin (Str, 10 μg/ml) was added to the cultures of *D. radiodurans* mutant strains.

### Construction of *D. radiodurans* Knockout Mutant

Mutant was constructed by double crossover recombination of a streptomycin resistance cassette into the genome as described previously (Tian et al., 2007). Briefly, upstream and downstream fragments of the target gene flanked by *Bam*HI and *Hind*III, respectively, were PCR-amplified with the corresponding primers listed in Supplementary Table S2. After digestion, these fragments were ligated to a streptomycin resistance cassette and transformed into the competent cells using the CaCl_2_ method. Mutants were screened on TGY plates containing streptomycin, and then all the recombinants were confirmed by PCR analysis and sequencing.

### Cell Growth Inhibition and Viability Assays

The toxin and antitoxin homology genes were cloned into pBAD33 and pET28a, the resulting plasmids were transformed or co-transformed into *E. coli* BW25113Δ6 cells, and their expression was induced by arabinose and isopropyl-β-D-thiogalactoside (IPTG), respectively. Cultures were grown to an optical density at 600 nm (OD_600)_ of 0.6, then 0.2% arabinose and/or 0.3 mM IPTG were added to induce the expression of the toxins and/or antitoxins. *E. coli* cells transformed with pBAD33-*dr0417* and pET28a-*dr0416*, or pBAD33-*dr0662* and pET28a-*dr0661* were cultured overnight in LB broth. Then, the cultures were diluted into fresh LB broth and grown to OD_600_ of 0.55. Subsequently, each culture was divided into four equal parts. One of the cultures served as control, to which was added equal ddH_2_O, while 0.2% arabinose, 0.3 mM IPTG or both 0.2% arabinose and 0.3 mM IPTG were added to the other three cultures to induce the expression of toxin and/or antitoxin. These cultures were incubated at 37°C and the OD_600_ was measured hourly to evaluate the cell growth.

The viability of *D. radiodurans* exposed to DNA damage stresses was determined as described previously (Tian et al., 2007). Cells grown to an OD_600_ of 0.6 were harvested and suspended in sterile phosphate-buffered saline (PBS). For the MMC (Sigma Co., United States) treatment, the cells were treated with 5–20 μg/ml MMC, and samples were taken after 40 min and plated on TGY agar plates after being diluted appropriately. For the H_2_O_2_ treatment, the cells were treated with different concentrations of H_2_O_2_ for 30 min. After incubation, the reaction was stopped by catalase (20 U) for 15 min. Then, the cells were diluted and plated on TGY agar plates. For the γ-radiation treatment, cell suspensions were irradiated at room temperature for 2 h with ^60^Co γ-ray (point source, Zhejiang Academy of Agricultural Sciences, Zhejiang, China) at several different doses (from 3 to 12 kGy), which were adjusted by changing the distance of samples from the γ-ray source (Wang et al., 2008). After radiation, the cells were diluted appropriately with PBS and plated on TGY agar plates. All plates were incubated at 30°C for 3 days to count the viable colonies. All the tests were repeated three times independently. The viable fractions were expressed as the logarithm of the ratio of the number of colonies from the treated samples to those from the untreated control.

### Protein Expression and Purification

DR0416 and DR0417 were expressed from a pET28a+ vector containing a N-terminal 6×His tag in BL21 (λDE3) pLysS cells. When cells grown in LB media reached an OD_600_ of 0.6–0.8, protein expression was induced with 0.3 mM IPTG at 30°C for 5 h. Then, cells were pelleted by centrifugation and re-suspended in lysis buffer A [20 mM pH 8.0 Tris-HCl, 500 mM NaCl, 5% (v/v) glycerol] containing PMSF (phenylmethanesulfonyl fluoride, Roche Biochemicals, Switzerland) and lysed by sonication. Cell lysates were centrifuged at 15,000 × *g* for 30 min to remove debris, and the supernatant was loaded onto a Ni-NTA column (GE Healthcare, United States), the target proteins were eluted with elution buffer B (20 mM Tris–HCl pH 8.0, 500 mM NaCl, and 500 mM imidazole). The collected protein fractions were dialyzed against buffer C [20 mM Tris-HCl pH 8.0, 100 mM NaCl, 5% (v/v) glycerol] and then loaded onto HiTrap Q ion exchange column (GE Healthcare) and eluted by gradient elution. Finally, proteins were further purified on a Superdex 75 column (GE Healthcare) with buffer C. Fractions containing the target proteins were pooled, concentrated, flash-frozen in liquid nitrogen, and stored in -80°C.

### Extraction of Total RNA from *D. radiodurans*

Total RNA of *D. radiodurans* was isolated using the TRIzol method according to the manufacturer’s protocol (Ambion, United States). A culture of *D. radiodurans* was grown in TGY medium until the OD_600_ reached ∼0.6. Then, the cells were pelleted and resuspended in 1 ml of TRIzol solution. After vortexing, the samples were incubated for 15 min at room temperature.

### *In Vitro* RNA Cleavage Activity of MazF-dr

Purified DR0417 was incubated with *D. radiodurans* total RNA or MS2 phage RNA (RefSeq accession no. NC_001417) at 37°C for 15 min. The reaction mixture consisted of DR0417, RNA, 0.1 mM ethylenediaminetetraacetic acid (EDTA) and 0.5 μl of RNase inhibitor (Promega, United States) in 20 mM Tris-HCl (pH 8.0), with or without DR0416. After denaturation in urea, the mixtures were loaded onto a 4.5% polyacrylamide gel containing 8 M urea, electrophoresed, and then stained with ethidium bromide (EB).

### Synthesis of 16S rRNA

*Deinococcus radiodurans* 16S rRNA was transcribed *in vitro* using the T7 promoter method (Hook-Barnard et al., 2007). Briefly, 16S rDNA fragments used for the transcription reaction were amplified with the primers 16S-TR and 16S-TS that contain the T7 promoter sequence (Supplementary Table S2). After purified, *in vitro* transcription reaction was carried out at 37°C for 1.5 h with 50 U T7 RNA polymerase (New England Biolabs, United Kingdom). Then DNase I (New England Biolabs) was used to digest the 16S rDNA substrate. The obtained 16S rRNA products were extracted using phenol-chloroform and precipitated with ethanol.

### Primer Extension Assays

For a primer extension analysis of the cleavage sites of MazF-dr, the 16S RNA was digested with MazF-dr at 37°C for 10 min. The reaction mixture contained 1 μg of 16S RNA substrate, 0.05 μg of MazF-dr and 0.5 μl of RNase inhibitor in 20 mM Tris-HCl buffer (pH 8.0). Primer extension was carried out in 10 μl of the reaction mixture containing 5′-FAM labeled primers (16S-F1, 16S-F2, and 16S-F3 as shown in Supplementary Table S2) at 42°C for 1 h, and then it was stopped by adding 2 μl of quench loading buffer (95% formamide, 20 mM EDTA, 0.05% bromophenol blue, and 0.05% xylene cyanol EF). The samples were incubated at 70°C for 5 min prior to electrophoresis on a 6% sequencing gel. The four sequence markers were produced using PCR with 16S DNA as template and the same primers that were used in the primer extension assay. The PCR mixture contained 1 μg of 16S DNA substrate, 0.05 mM ddNTPs, 0.5 mM dNTP, therminator DNA polymerase (New England Biolabs) and 1 mM FAM labeled primer. A sequence logo plot of multiple sequence alignment was generated by the Weblogo online program to analyze the conserved RNA cleavage sites of MazF-dr^2^ (Doerks et al., 2002).

### Cleavage Activity of MazF-dr Using Synthesized FAM-Labeled RNAs

Oligoribonucleotides labeled with 5′-FAM were commercially synthesized by Takara Bio (Japan). The endoribonuclease activity of MazF was assayed in a 10 μl reaction mixture containing MazF, FAM-labeled RNA substrates, 0.2 μl of RNase inhibitor (Promega, United States), 20 mM Tris-HCl (pH 8.0), and 0.1 mM EDTA. The reaction mixture was incubated at 37°C for 15 min, and stopped by adding 2 μl of RNA loading buffer (0.25% bromophenol blue, 0.25% xylene cyanol EF, and 15% Ficoll). Then, the reaction mixture was loaded onto a 20% urea PAGE and scanned by the Typhon system (GE Healthcare, United States).

### Confocal Microscopy Assay

The cell death was assessed by using confocal microscopy. The cells were stained using the Live/Dead Kit containing PI and SYTO 9 (Invitrogen). Cells grown to an OD_600_ of 0.8 were washed twice with 0.85% NaCl. Cells treated with a sub-lethal dose of MMC (15 μg/ml, 40 min), as well as untreated cells, were collected by centrifugation. Then the cells were stained using 1 μl of a 1:1 mixture of PI and Syto 9 from the kit and were incubated for 15 min at room temperature keeping in the dark. Following the wash by 0.85% NaCl twice, 100 μl of 4% formalin was applied for the fixation. The cells were washed twice with PBS buffer and re-suspended in 20 μl of 50% glycerol. A confocal microscopy assay was performed using a Leica DM4000 fluorescence microscope. Living cells were stained by Syto 9 (green) and death cells were stained by PI (red).

### Transmission Electron Microscopy (TEM)

For the TEM analysis, *D. radiodurans* cells treated with MMC (15 μg/ml, 40 min), as well as untreated cells, were collected by centrifugation. Samples were processed as described previously (Rothfuss et al., 2006). The cells were fixed with 2.5% glutaraldehyde in phosphate buffer (pH 7.0) overnight and then embedded in 5% agar. Thin sections of the samples were stained with uranyl acetate for 15 min and observed by TEM (JEM-1230, JEOL, Japan).

## Author Contributions

TL, BT, and YH were responsible for the experiments design and drafted the manuscript. XM revised the manuscript finally. SD and YJ constructed the vectors and mutants. The viability assays were completed by ML. JL and JY performed the primer extension assays. TL performed the confocal microscopy and TEM observation and all data analysis. All authors reviewed the manuscript and approved the version to be published.

## Conflict of Interest Statement

The authors declare that the research was conducted in the absence of any commercial or financial relationships that could be construed as a potential conflict of interest.

## Footnotes

1 https://www.ncbi.nlm.nih.gov/

2 http://weblogo.berkeley.edu/logo.cgi
