# Supplementary material for: Deinococcus radiodurans Toxin–Antitoxin MazEF-dr Mediates Cell Death in Response to DNA Damage Stress
Source: Front Microbiol. 2017 Jul 26;8:1427. doi: 10.3389/fmicb.2017.01427 (PMC5526972; doi:10.3389/fmicb.2017.01427)
Supplement: Supplementary file 1 [file Data_Sheet_1.DOCX]

***Supporting information***

***Deinococcus radiodurans* toxin-antitoxin MazEF-dr mediates cell death in response to DNA damage stress**

Tao Li ^1^, Yulan Weng ^1^, Xiaoqiong Ma ^2^, Bing Tian ^1*^, Shang Dai ^1^, Ye Jin ^1^, Mengjia Liu ^1^, Jiulong Li ^1^, Jiangliu Yu ^1^, Yuejin Hua ^1*^

^*^*Corresponding author*: Bing Tian (tianbing@zju.edu.cn); Yuejin Hua (yjhua@zju.edu.cn), Institute of Nuclear-Agricultural Sciences, Zhejiang University, No.268, Kaixuan Road, 310029 Hangzhou, China, Tel/Fax: +86-571-86971215

**Supplementary Figure 1.** Multiple sequence alignments of MazF homologs. Identical residues are shown as white letters with black background, and similar residues are shown as black letters with a gray background. Sequences were from several bacteria, including EcMazF from *E. coli*, CdMazF from *Clostridium difficile*, BsMazF from *Bacillus subtilis*, SaMazF from *Staphylococcus aureus*, MtMazF from *Mycobacterium tuberculosis*, and DR0417 and DR0662 from *D. radiodurans*. DR0417 shows several conserved residues that are required for substrate binding and toxin activity. The image was plotted by BOXSHADE 3.21 (http://embnet.vital-it.ch/software/ BOX_form.html).

**
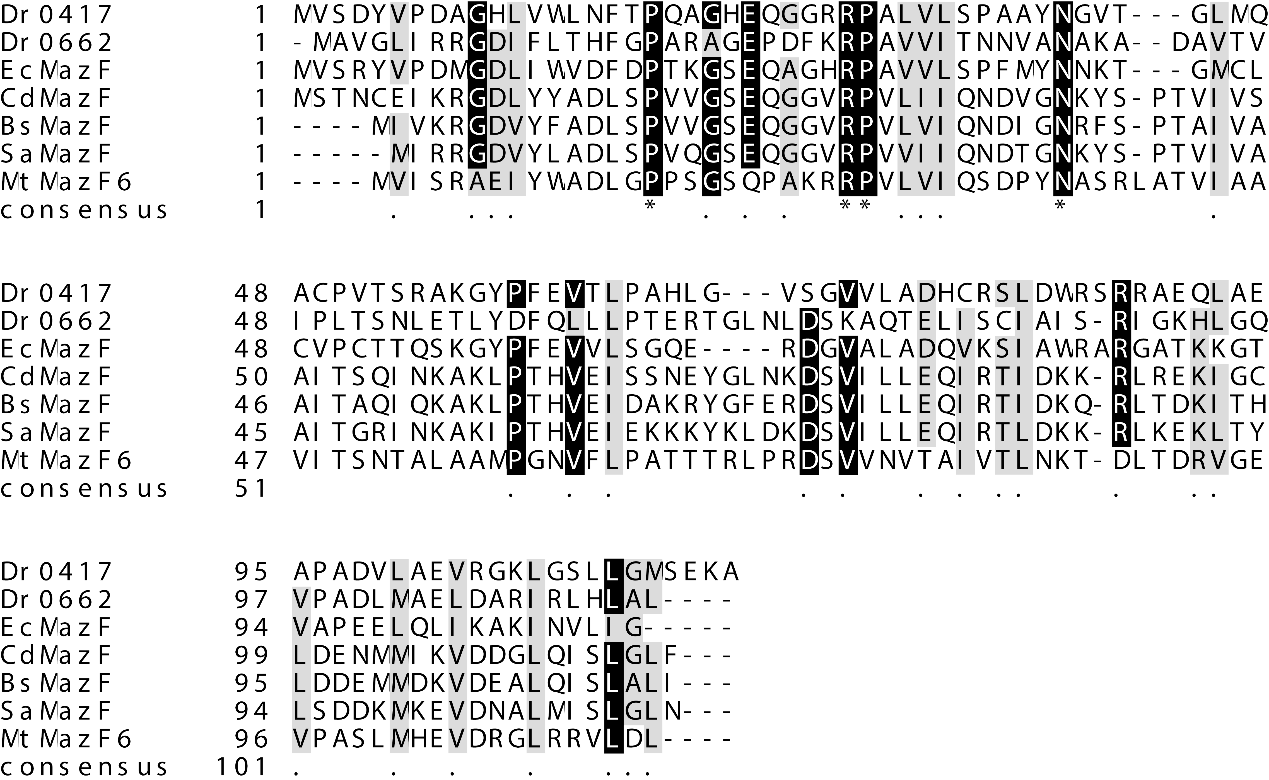
**

**Supplementary Figure 2.** Western bolt assays of DR0417 and DR0662 over-expressed in *E. coli*. Protein samples separated by SDS-PAGE were transferred to PVDF membranes. The membranes were incubated in PBS containing 0.1% Tween-20 (PBST) and 5% skim milk powder for 90 min at room temperature, and then they were washed five time using PBST. Rabbit anti-DR0417 and anti-DR0662 polyclonal antibodies, obtained using purified proteins as antigen, were applied to detect the proteins. Goat anti-rabbit IgG conjugate HRP (Sigma, USA) was added as the secondary antibody and detected using a colorimetric reaction. Lane 1: *E. coli* with empty vector. Lane 2: un-induced *E. coli* cells transformed with *dr0417* or *dr0662*. Lane 3: 0.2% arabinose-induced *E. coli* cells transformed with *dr0417* or *dr0662*.

**
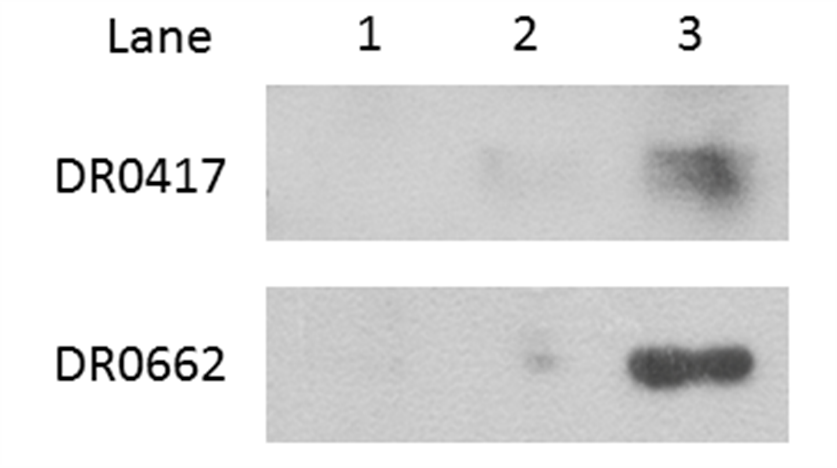
**

**Supplementary Figure 3.** Construction of *mazEF-dr* knock-out mutant. (A) Schematic representation of the gene mutation by homologous recombination which replaced the *dr0416-dr0417* operons with streptomycin-resistant cassette. P1, P2, P3, and P4 refer to the primer pairs (Table S2). (B) Electrophoresis analysis of PCR products to verify the mutation of *dr0416-dr0417* loci. DNA fragments across the *dr0416-dr0417* loci were amplified. Lane 2 and lane 3, DNA amplicons amplified with primer pair P1 and P4 in the mutant and the wild type, respectively. The corresponding amplicon (lane 2, 1803bp) from the mutant is 419 bp larger than the amplicon (lane 3, 1384bp) from the wild type, indicating that *dr0416-Dr0417* was replaced with the streptomycin-resistance cassette (927 bp). Moreover, interspace DNA fragments from the wild type and the mutant were amplified (lane 4 and lane 5), respectively. No products corresponding to the size of the fragment from wild type (lane 5) was observed in the mutant (lane 4), suggesting that the wild type alleles had completely segregated in the mutant. Lane 1, Marker.


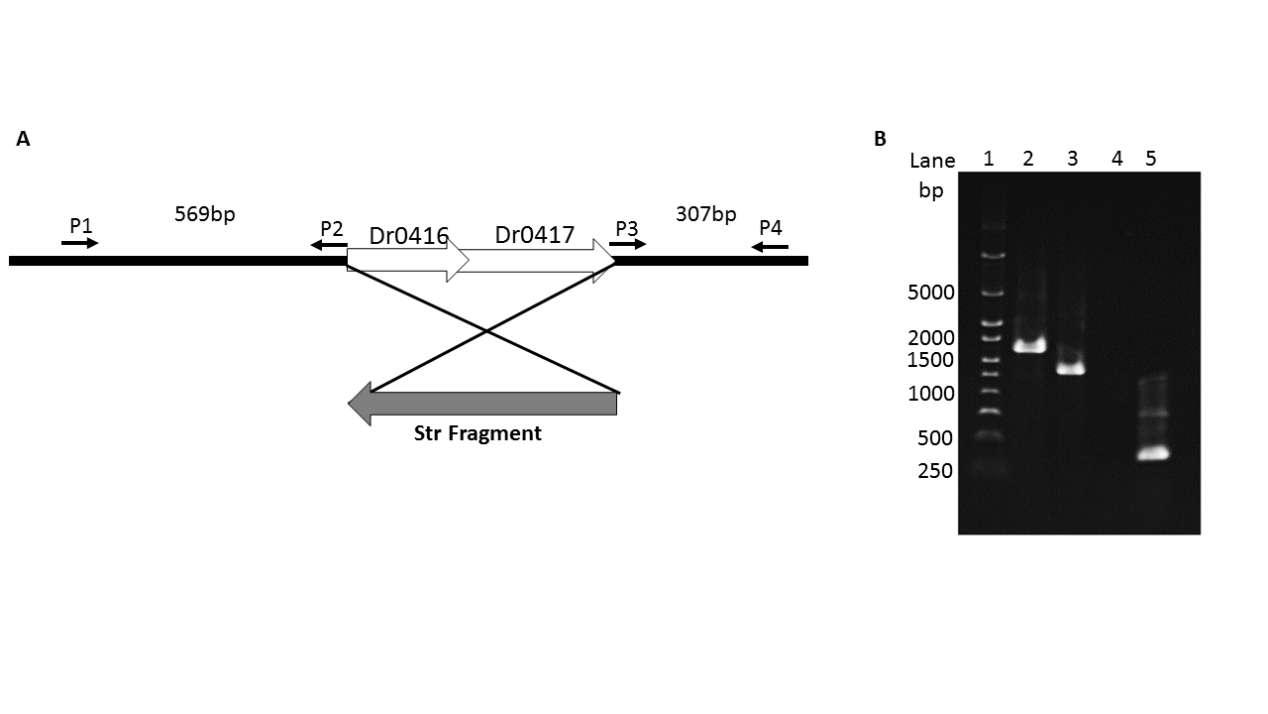


**Supplementary Figure 4.** The *mazE-dr* and *mazF-dr* are co-transcribed. (A) An overview of the *mazEF-dr* operon construct indicating the positions of the amplicons. Total RNA of *D. radiodurans* wild type and ΔMazEF-dr were extracted, then reversely transcribed into cDNA. Primers were used to amplify the fragments within the *mazEF-dr* operon. (B) PCR analysis of the amplicons from the wild type indicated that the *mazE-dr* and *mazF-dr* are co-transcribed. Lane 1, DNA marker; Lane3 and lane 5, amplicons from the wild type using P1-1 and P1-2, P2-1 and P2-2, respectively. Lane2 and lane 4, amplicons from ΔMazEF-dr using P1-1 and P1-2, P2-1 and P2-2, respectively.


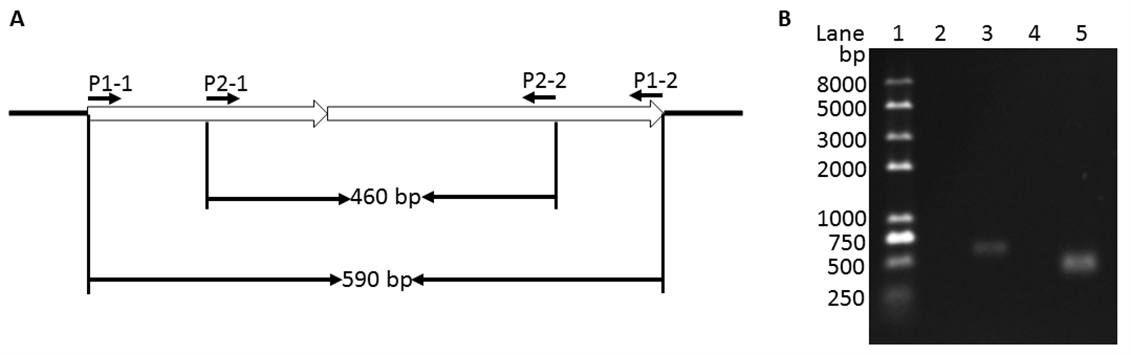


**Supplementary Figure 5.** MazE-dr can bind to the promoter of *mazEF-dr* operon. 200 ng promoter DNA was incubated with or without MazE-dr. Lane 1: DNA without protein added. Lane 2: DNA was incubated with 100 nM MazF-dr. Lane 3-6: DNA was incubated with MazEF-dr complex at an increasing concentration from 1 to 100 nM. MazEF complex was purified by sieve chromatography following the method as described by Kaiying Cheng, *et al* (J. Bacteriol, 2015, 197(12):2048-61). Lane 7-9, DNA was incubated with MazE-dr alone at an increasing concentration from 10 to 100 nM. The additional bands appeared in line 7-9 might represent the DNA complexes formed with MazE oligomers. DdrO from *D. radiodurans* was incubated with the DNA as a control. The reaction mixture was separated by gel electrophoresis and stained by EtBr.


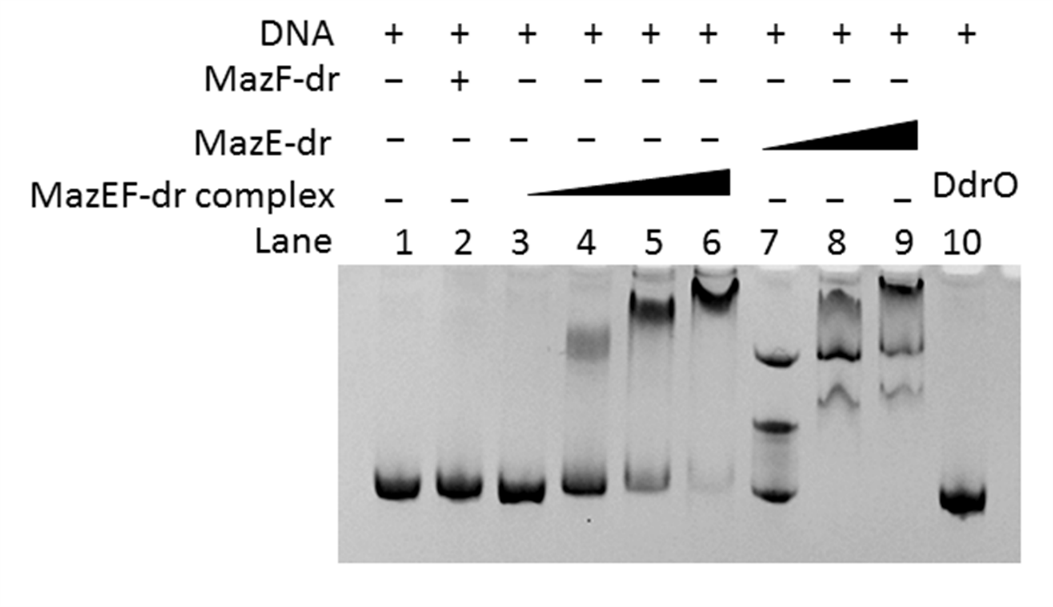


**Supplementary Figure 6.** Quantitative real-time PCR (QRT-PCR) assays of selected DNA repair related genes in *D. radiodurans* wild type and ΔmazEF-dr under 15 μg/ml MMC treatment. **Black filled**: the wild type treated with 15 μg/ml MMC for 40 min compared to untreated wild type; **Red filled**: ΔmazEF-dr treated with 15 μg/ml MMC for 40 min compared with untreated ΔmazEF-dr; **Blue filled**: untreated wild type compared to untreated ΔmazEF-dr. Relative fold change of transcription is expressed as 2^-ΔΔCt^. All assays were performed using the STRATAGENE Mx3005P™ real-time detection system, following the method described previously by Lin lin, *et al* (Molecular Microbiology, 2016, 100(3): 527-541).

**

**

**Supplementary Table 1. List of strains and plasmids used in this study.**

| Strain or plasmid | Description or information | Reference and/or resource |
| --- | --- | --- |
| Strains |  |  |
| *E. coli* |  |  |
| BL-21(λDE3), pLysS | *F^-^ ompT hsdSB (rB - mB -) gal dcm*  *λ(DE3) pLysS Camr* | TransGen Biotech |
| DH5ɑ | *F-φ80 lac ZΔM15 Δ (lacZYA-arg F) U169 endA1 recA1 hsdR17 (rk^-^, mk^+^) supE44λ- thi -1 gyrA96 relA1 phoA* | TransGen Biotech |
| BW25113Δ6 | *lacI^q^ rrnB_T14_Δlac-Z_WJ16_ hsdR514 ΔaraBAD_AH33_ ΔrhaBAD_LD78_ΔchpBIK* *ΔdinJ-yafQ ΔhipBAΔmazEF ΔrelBE ΔyefM-yoeB* | A kind gift from Professor Nancy A. Woychik |
| *Deinococcus radiodurans* |  |  |
| R1 | *D. radiodurans* wild type | Laboratory Collection |
| ΔMazEF | Disruptant of R1 deleted *dr0416 and dr0417*, Strr | This study |
| *pprI* mutant | Disruptant of R1 deleted *pprI* (DR_0167), Kanr | Laboratory Collection |
| *crtB* mutant | Disruptant of R1 deleted *crtB* (DR_0862), Kanr | Laboratory Collection |
| Plasmids |  |  |
| pET28a+ | Expression vector with strong T7  promotor, His-tag | Novagen |
| PRADK | *E.coli* and *D. radiodurans* shuttle vector | Laboratory Collection |
| pBAD-33 | Arabinose inducible vector | [[1](#_ENREF_1)] |

1. Guzman, L., *et al.*, Tight regulation, modulation, and high-level expression by vectors containing the arabinose PBAD promoter. *Journal of Bacteriology*, 1995. **177**(14): 4121-4130.

**Supplementary Table 2. Primers used in this study.**

| Primer name | Primer sequence (5’ to 3’) | label |
| --- | --- | --- |
| **Mutant construction** | | |
| 0416-0417-P1 | CACCGCCAGAAACCCGTAGAGC |  |
| 0416-0147-P2 | CGGGATCCAAAGATACATTTCCTGAC |  |
| 0416-0147-P3 | CCAAGCTTCCGAACAACTTGCGGA |  |
| 0416-0147-P4 | GTCCCAATCCAGCGGGTGAAGG |  |
| 0416-0417-c-f | GAGTCAAATTCAGAAATGGGGCAACAG |  |
| 0416-0417-c-r | TCCAAGGTGAGCAGGGAGCGTC |  |
| **Protein expression** | | |
| 0417-EF | TTCATATGGTAAGCGATTATGTCCCGG |  |
| 0417-ER  0416-EF | TTGGATCCGTCACTCACTGTTCACTT  TTCATATGACGAGTCAAATTCAGAAATGGG |  |
| 0416-ER | TTGGATCCGTCCGGGACATAATCGCTTA |  |
| 0662-EF | TTCATATGGCTGTAGGACTCATCCGG |  |
| 0662-ER | TTGGATCCTTACAGGGCAAGGTGAAGG |  |
| 0661-EF | TTCATATGACTTACCAGAACGCCGAA |  |
| 0661-ER | TTGGATCCTACAGCCATTCGCTACCGTC |  |
| 0417-pBAD-F | CCCCCGAGCTCGGATCCAAAATAAGGAGGAAAAAAAAATGGTAAGCGATTATGTCCCG |  |
| 0417-pBAD-R | CCCCCAAGCTTGAATTCTCATGCCTTCTCGGACATG |  |
| 0662-pBAD-F | CCCCCGAGCTCGGATCCAAAATAAGGAGGAAAAAAAAATGGCTGTAGGACTCATCCGGC |  |
| 0662-pBAD-R | CCCCCAAGCTTGTGGCTCGTCCCCTTCGACTGTTTA |  |
| **Primer extension** | | |
| 16S-TS | AGATCTCGATCCCGCGAAATTAATACGACTCACTATAGGGGATTTATGGAGAGTTTGATCCTGGCTC |  |
| 16S-TR | AAAGGAGGTGATCCAACCGCACCTT |  |
| 16S-F1 | CACATCACGTATTAGCGGACCTTTC | 5’FAM |
| 16S-F2 | CTAAGGCTCTTTCGTCCCAGATTCA | 5’FAM |
| 16S-F3 | AGGAGGTGATCCAACCGCACCTT | 5’FAM |
| **MazF-dr cleavage activity assay** | | |
| FR1 | GUCGUAACAAG | 5’FAM |
| FR2 | GUCGUGACAAG | 5’FAM |
| FR3 | GUCGUAUCAAG | 5’FAM |
| FR4 | GUCGUAGCAAG | 5’FAM |
| FR5 | GUCGUAAGAAG | 5’FAM |
| FR6 | GUCGUAACGAG | 5’FAM |
| FR7 | GUCGUUACAAG | 5’FAM |
| FR8 | GUCGUCACAAG | 5’FAM |
| **QRT-PCR** | | |
| recA-s-RT | ACCGACGCCAAGGAACGCAGCAA |  |
| recA-a-RT | TCAGAGCAGCCACCGAGTCCACGAC |  |
| ddrO-s-RT | TCAAGGACGTGGCCGAGGTTGC |  |
| ddrO-a-RT | TTCAGGATGCGTTTGAGATGCAGGTAG |  |
| pprI-s-RT | GGGGTGCCCGGCGTAGACCTCAAAT |  |
| pprI-a-RT | TTGCCCGGCGCACAGACCGCGTAGATGA |  |
| recJ-s-RT | GCGGCAGAACTTGCCCTCAACG |  |
| recJ-a-RT | GTCTTCGGTCCCAGCGCAAACG |  |
| uvrC-s-RT | AAGCCAACCTCATCAAGCAGCACCG |  |
| uvrC-a-RT | GCCGCCACCTTCATGTCCTCTTTCA |  |
| pprA-s-RT | TCAGGGCATAATAAAGGCAGTATGGCAAGG |  |
| pprA-a-RT | TTGAGCTGCGCCAACGGCAAGTCG |  |
| radA-s-RT | CCTGCAAATGACCCGCGACACC |  |
| radA-a-RT | AGGGCCTGCACTTCCAGCAGCAT |  |
